# Towards Standardization in the Diagnostic Evaluation of ACL Injuries in Skeletally Immature Patients

**DOI:** 10.3390/ijerph18052684

**Published:** 2021-03-07

**Authors:** Liliana Seabol, Stephanie Boden, Max Herman, Ethan Ruh, Mininder Kocher, Michael McClincy

**Affiliations:** 1 Department of Orthopaedic Surgery, University of Pittsburgh School of Medicine, Pittsburgh, PA 15261, USA; lgs24@pitt.edu; 2 Department of Orthopaedic Surgery, University of Pittsburgh Medical Center, Pittsburgh, PA 15213, USA; bodensa2@upmc.edu (S.B.); ruher@upmc.edu (E.R.); 3 School of Health Sciences, Duquesne University, Pittsburgh, PA 15282, USA; hermanm2@duq.edu; 4 Boston Children’s Hospital, Boston, MA 02115, USA; mininder.kocher@childrens.harvard.edu

**Keywords:** ACL, pediatric, sports medicine, skeletally immature

## Abstract

The purpose of this study was to establish consensus regarding a standardized approach to the diagnostic evaluation of ACL tears in pediatric/adolescent patients. Despite an abundance of literature evaluating management techniques, no standardized consensus exists regarding evaluation in these patients. A three-step classic Delphi technique was employed. The panel included 12 Orthopaedic Sports Medicine specialists from across the United States with training in pediatric and adult ACL injuries. Panelists were presented with four clinical vignettes. Consensus was established if ≥66% of respondents reached agreement. Across all four rounds of this study, 100% participation was achieved, and consensus was reached for a majority of diagnostic domains. For history, previous injuries, sports participation, and current symptoms were endorsed for all vignettes. The consensus radiographic sequences across all four vignettes included: standing AP, flexion (tunnel or notch view), lateral, long-leg alignment, and bone age (left hand) views. Radiographic interpretation responses met consensus with interpretations were split by gender. Cross-sectional imaging met consensus with 100% support for MRI. In this Delphi study, we identified a standardized diagnostic treatment approach derived from expert opinion applicable to all skeletally immature patients with ACL tears, which can serve as a framework for evaluation to aid clinical decision making.

## 1. Introduction

The incidence of pediatric anterior cruciate ligament (ACL) tears has dramatically increased over the past two decades, likely in part due to increased participation in youth sports, early sports specialization, increasing intensity of youth sport training, and increased awareness among physicians [1,2,3,4]. Recent epidemiological studies have demonstrated a steady increase in rates of pediatric ACL tears, with the number of ACL tears reported increasing by approximately 2.3% annually [5,6]. While historically pediatric ACL tears have been managed nonoperatively until skeletal maturity due to concerns regarding physeal damage associated with traditional ACL reconstructions, more recent literature has highlighted the perils of nonoperative and delayed operative management on pediatric and adolescent knees [7,8,9,10,11].

As the prevalence of ACL injuries in the skeletally immature patient population has risen, the number of ACL reconstructions (ACL-R) performed in this population has also drastically increased. In fact, pediatric and adolescent patients now represent the largest per capita demographic for ACL reconstructions [1]. Skeletally immature patients with ACL injuries also present a unique challenge for treating surgeons due to the presence of open physes and the potential growth disturbances that can arise in cases of iatrogenic injury during surgical reconstruction [12]. Skeletally immature patients can undergo reconstruction using techniques which minimize damage to the physes. Such options include the iliotibial band intra- and extra-articular reconstruction, over-the-top femoral, and all-epiphyseal tunnel techniques. [12,13] Despite the numerous studies evaluating reconstruction management techniques for pediatric ACL tears [13,14,15,16,17], to date there is no standardized consensus regarding the diagnostic work-up of ACL tears in skeletally immature patients. In 2014, the American Academy of Orthopaedic Surgeons (AAOS) developed a Clinical Practice Guidelines (CPG) for the management of ACL injuries, but the guidelines were not specifically targeted towards ACL injuries in skeletally immature patients [18]. Given the unique anatomy and risk of associated iatrogenic injuries found within the skeletally immature athlete population, it is crucial to develop a standardized approach to the clinical assessment of skeletally immature patients with ACL tears.

In this study, we utilized the Delphi method to assess clinical evaluation methods used amongst experts in the field of pediatric and adult ACL-R. The purpose of this study was to establish consensus regarding a standardized approach to the diagnostic evaluation of ACL tears in the pediatric and adolescent patient population.

## 2. Materials and Methods

### 2.1. Delphi Panel

The expert panel in this study consisted of twelve orthopedic surgeons with extensive experience and dedication to the treatment of ACL injuries. They were purposefully sampled from geographically disparate institutions spread throughout the United States. Participants were selected based on multiple criteria, including (1) extensive clinical expertise in ACL reconstructive surgeries defined as >5 years clinical practice and >50 ACL-R per year and (2) research expertise in ACL injury management defined as >5 first or senior author publications in National Library of Medicine (NLM) indexed journals. A dedicated effort was made to select specialists from both pediatric and adult sports medicine practices. All members consented to participate in this IRB exempted study, and participants were blinded to each other for the entire duration of the study.

### 2.2. Delphi Structure and Data Collection

A three-step classic Delphi method was used to establish consensus techniques in the diagnostic evaluation of pediatric ACL injuries [19]. Consensus was defined a priori as ≥66%, which is relatively low per standard Delphi methods but was purposefully chosen to account for the expected levels of disagreement within our diverse expert panelist group. Definitions of consensus level are commonly based on accepted standards such as voting percentages (simple majority, two-thirds majority, absolute majority) and we felt that a two-thirds majority would be most appropriate for this study [20]. Our study had the dual objective of achieving consensus and, equally importantly, understanding areas where consensus could not be reached and reasons for disagreement.

**Figure 1:**
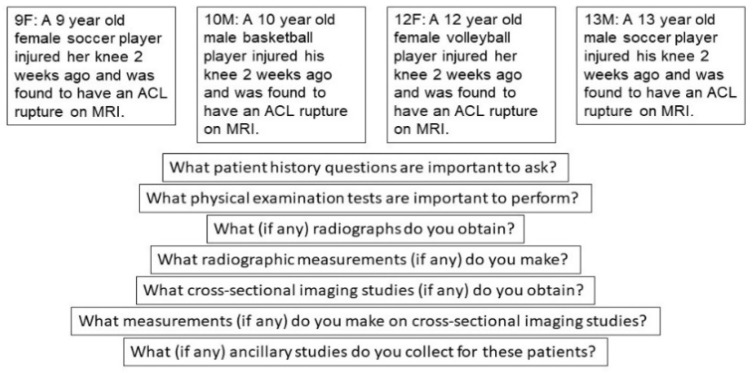
Case vignettes provided to panelists in Delphi study to evaluate management of pediatric ACL injury.

Delphi panelists were presented with four clinical vignettes representing a spectrum of pediatric and adolescent ACL injury patients (Figure 1).

Based on these clinical vignettes, panelists were presented with three iterative survey rounds. Questionnaires for rounds 1–3 were distributed online via an emailed link. Individual follow-up emails were used to gain responses when participants did not respond to the standard email prompts. Delphi participants remained anonymous to all participants but the study coordinators, and responses were de-identified during thematic content analysis. For each survey round, thematic content analysis of participants’ responses was completed by two study members. Any disagreements were resolved by a third team member.

In round 1, panelists were presented with four clinical vignettes, and for each vignette, seven open ended questions regarding their clinical practice habits across seven aspects of clinical diagnostics:

1. What patient history questions are important to ask?
2. What physical examination tests are important to perform?
3. What (if any) radiographs do you obtain?
4. What radiographic measurements (if any) do you make?
5. What cross-sectional imaging studies (if any) do you obtain?
6. What measurements (if any) do you make on cross-sectional imaging studies?
7. What (if any) ancillary studies do you collect for these patients?

The panelists were prompted to provide free-text responses to each.

Panelists provided detailed descriptions of their routine evaluation of the patients described in the vignettes. Responses were collected and coded for common thematic content. Responses reported by ≥50% of panelists were considered modal, while responses reported by ≥25% of panelists formed a second tier of responses.

In round 2, panelists were prompted with the same four vignettes and seven questions as round 1. In addition, they received the modal (≥50%) and second tier (≥25%) responses from the round 1 surveys. Panelists were asked to “agree” or “disagree” with the modal response for each vignette/question and suggest additions and/or subtractions to the modal response. In cases where panelists chose to provide additional components to the modal response, they were presented with the second-tier responses as options from which to choose and were also permitted free-text additions. Resulting responses were again coded for thematic content and modal responses were adjusted as appropriate. Two questions from each vignette achieved unanimous consensus following round 2, so these questions were omitted from round 3.

In round 3, panelists again received the same vignettes and five of the seven questions, as well as modal and second-tier responses from round 2. Analysis of the third-round data provided options for which consensus had been gained as well as rationale for disagreement.

## 3. Results

### 3.1. Delphi Panelists

Twelve orthopedic surgeons with expertise in the treatment of pediatric and adult ACL injuries were included in this study. Six panelists primarily practiced at adult facilities and six panelists primarily practiced at pediatric facilities. Overall, 100% participation was achieved with all 12 experts completing all three Delphi survey rounds.

### 3.2. Consensus and Disagreement

Table 1, Table 2, Table 3 and Table 4 display the levels of agreement and consensus achieved at the completion of round 3. Agreement was defined as group acceptance (≥66%) of an individual component of the modal response, while consensus was defined as group acceptance (≥66%) of the entire modal response. Response components are presented individually for each vignette, but responses reaching agreement across all vignettes are noted. Sources of disagreement are also noted.

#### 3.2.1. Historical Factors

All vignettes achieved consensus for historical features to be scrutinized during patient evaluation (Table 1). The commonly agreed upon topics for each vignette included injury history, sports participation, and current symptoms. Family history was included for all vignettes but the adolescent female, but a minority of providers also noted family history to be relevant in this vignette. The adolescent female vignette also included an extra inquiry about menarche in the modal response.

#### 3.2.2. Physical Examination Factors

All vignettes, except 10M, achieved consensus for a list of examination techniques: Lachman, pivot-shift, collateral exam, meniscal exam, ROM, and ligamentous laxity (Table 2). Additional tests of effusion, anterior and posterior drawer, alignment, gait, and patellar examination were a source of controversy across the vignettes. Effusion was included in the consensus response for all vignettes but the 13M, in which case it received minority support. Vignettes stated that evaluation took place at 2 weeks following injury, but panelists made no specific further mention of physical or other assessment within a specific time frame. The 10M vignette did not achieve consensus in spite of high agreement with all components because a number of experts endorsed additional examination techniques, all with limited support from other experts. 

#### 3.2.3. Radiographic Sequences

All vignettes generated a consensus radiographic sequence including standing AP, PA flexion (45 or tunnel), lateral, and long-leg alignment views of the lower extremities, as well as a left hand film to ascertain bone age (Table 3a). The flexion view, long-leg alignment view, and bone age films provided the greatest controversy during early survey rounds, but the majority of experts ultimately supported their inclusion for all vignettes consensus statements.

#### 3.2.4. Radiographic Measurements

All vignettes garnered consensus with tibial slope measurement on lateral radiographs (Table 3b). The preadolescent and adolescent male vignettes also included assessment of physeal status in their consensus responses, which can be interpreted on any image. In initial surveys, several experts included physeal status in all four vignettes. In subsequent rounds, while it maintained support in the two male vignettes, it did not sustain enough support for inclusion in the female vignettes. Multiple experts endorsed mechanical axis measurement for all vignettes, but it did not reach the threshold for modal response inclusion. Other radiographic evaluations, such as ACL-specific measurements and the coronal angle, were sources of controversy, with few experts endorsing their inclusion.

#### 3.2.5. Cross-Sectional Imaging

Cross-sectional imaging was the greatest source of agreement for the experts, with 100% endorsing support for MRI after round 2. As such, the question was removed from the round 3 survey. Even in the initial survey round, most experts indicated their preference for MRI, and no alternative imaging modalities were endorsed.

#### 3.2.6. Cross-Sectional Measurements

No cross-sectional measurement met the threshold for inclusion into the modal response, and consensus (no measurements) was achieved for all vignettes. Several respondents advocated for measurements such as ACL size/angle, tendon measurements, epiphyseal height, bone bruise measurements, and notch width across all vignettes. These measures failed to meet the threshold for agreement in any vignette. Each of these measurements led some experts to reject the modal response, yet the overall group was still able to meet the threshold for consensus.

#### 3.2.7. Ancillary Diagnostics

There was no commonly suggested ancillary study for any vignette. The consensus response for all vignettes was that no other ancillary testing is required. One expert was unwilling to agree with the modal response, but did not provide additional suggestions, indicating that his choices would depend on the specific patient. Ancillary diagnostics was also omitted from round 3, as overwhelming consensus (92%) had been reached following round 2.

## 4. Discussion

ACL tears are increasingly recognized in skeletally immature patients. Due to the increases in injury rates and shifts in management paradigms, more ACL-R procedures are being performed in skeletally immature athletes by a wider range of providers. Numerous studies have demonstrated that ACL-R surgical techniques and instrumentation must be approached with efforts to both resolve the ligamentous injury and avoid iatrogenic injury to open physes [15,21]. While understanding ACL-R techniques to avoid physeal injury is crucial, obtaining sufficient presurgical data to inform surgical decision making is of equal importance.

In this study, we utilized the Delphi method to develop a consensus diagnostic approach to skeletally immature athletes with ACL injuries from 12 orthopaedic sports medicine specialists from across the United States with various training and practice backgrounds. Across all four rounds of this study, 100% participation was achieved, and consensus was reached for a majority of the diagnostic domains employed in the evaluation of pediatric ACL tears. While we presented four clinical vignettes to provide a variety of patient scenarios, the ultimate goal of the study was to develop a standardized approach to the diagnostic evaluation of *all* pediatric patients with ACL tears. Experts were presented with only age, sex, mechanism of injury and MRI result of an ACL tear, and with this information were asked to establish a diagnostic approach for the skeletally immature patients.

In 2014, the American Academy of Orthopaedic Surgeons (AAOS) developed a Clinical Practice Guidelines (CPG) for the management of ACL injuries [18]. The AAOS guidelines recommend, in brief: a detailed historical summary of the injury, a comprehensive knee physical examination, two view plain radiography, and an MRI for a thorough preoperative evaluation. Our consensus diagnostic evaluation closely parallels much of the recommendations of the AAOS-CPG with a few notable additions with important consideration in skeletally immature athletes. As previously mentioned, these skeletally immature patients are often managed differently than their adult counterparts, and the differences in diagnostic evaluation reflect that.

Our consensus history added specific documentation of relevant family history of ACL injury in most vignettes, as well as documentation of menstrual history for the adolescent female vignette. Menarche has been endorsed in numerous studies as being associated with the deceleration of skeletal growth and progression towards skeletal maturity in females [2,3,4,5]. For physical examination, no exam techniques specific to the young athlete were included in the consensus physical examination for any vignette. Physical examination features of maturation, such as Tanner staging, was infrequently cited in the initial survey round but was never endorsed for inclusion in the consensus response. In the case of the 10M vignette, lack of consensus is likely due to the fact that while there was high agreement among experts for individual components of the physical exam, many experts had additional examination techniques that were not added by other experts, and never reached the threshold for inclusion in subsequent survey rounds, so were not offered as options for other experts to agree/disagree.

For preoperative imaging, the expert panelists achieved consensus agreement for the radiographic sequence consisting of standing AP, lateral, and flexion views (tunnel or notch view) of the knee, a long-leg alignment view, and bone age (left hand) view. This consensus sequence was adopted across all vignettes. This consensus added the flexion, long-leg alignment, and bone age radiographs to the AAOS-CPG, all of which are all important additions to the routine evaluation of a skeletally immature ACL injury. The notch view assists in evaluation of possible osteochondral defects, which have been reported in approximately 5% of skeletally immature patients at the time of ACL reconstruction [8]. Long leg alignment films identify pre-existing angular deformities that may contribute to increased graft strain and are important to follow over time to monitor for growth disturbances [22,23]. Bone age radiographs are traditionally assessed via a radiograph of the left hand, and assessment can help guide surgical decisions regarding operative approach, technique, and graft choice [24,25].

Radiographic interpretation also met consensus, although recommended interpretations were split by gender. All patients were recommended to undergo calculation of tibial slope, while only male patients were recommended to have a dedicated appreciation of physeal status (open versus closed). We hypothesize that this sex-based discrepancy existed as menarche status was heavily relied upon in the evaluation of female athletes. Interestingly, while experts endorsed long-leg alignment and bone age films in their consensus imaging sequences, the interpretations of “bone age” and “mechanical alignment/leg length” were only mentioned by a few experts for each vignette and did not meet the threshold for inclusion in the modal response. We suspect that their inclusion in the radiographic sequence modal response implied these respective measurements even though they were not expressly noted in the interpretation responses.

Cross-sectional imaging met consensus on the first survey round with 100% support for MRI in the imaging of these patients, paralleling the recommendations of the AAOS-CPG. Numerous experts endorsed various methods to interpret or quantify the MRI studies, however none garnered significant support. Ultimately, the consensus expert opinion was that no specific MRI interpretations were required for diagnosis.

In the majority of diagnostic domains, consensus responses were quite similar across the various patient vignettes. In no vignette or domain was a consensus threshold not met due to an excessively broad proposed approach. In other words, experts generally favored the most thorough approach towards evaluating these skeletally immature patients. As such, Table 4 presents the agreement responses (>67%) and most common “addition” responses not achieving agreement (<67%) across all vignettes. By applying the most expansive consensus response across all vignettes, all common sources of disagreement are resolved. The proposed standardized diagnostic approach to skeletally immature patients with ACL injuries is presented in the final column. The diagnostic evaluations most pertinent to our skeletally immature patients include: menarchal status (females) during patient history, addition of long-leg alignment (with mechanical axis/leg length interpretation) and bone age (with skeletal age interpretation) views to radiographic protocols, and interpretation of physeal status (open or closed) on knee radiographs. The remainder of the diagnostic evaluation closely parallels the AAOS-CPG for ACL injuries.

### Limitations

Due to the nature of this study, there are a few limitations that must be noted. As no prior standardized consensus exists regarding the diagnostic approach to ACL tears in the pediatric patient population, this study relied on the opinions of our expert panelists to generate the initial diagnostic approach. This technique may therefore introduce bias into the resultant modal responses; however, recruitment of a diverse expert panel may minimize this effect. Another limitation to the study was the use of an all-online survey process. Although panelists were asked to agree or disagree with changes after each round of questions, the lack of a face-to-face discussion may have hindered the ability to discuss and adapt various opinions. Alternatively, the lack of face-to-face conversation may have limited bias by a single stronger or more senior expert. Additionally, our panel consisted of only 12 experts, which may introduce sampling bias into the diagnostic preferences noted. This bias may be ameliorated by the use of a diverse expert panel of Sports Medicine specialists from across the United States, but a larger sampling of experts may alter the consensus rates. It is important to note that while the number of experts was limited to 12, thematic saturation was reached with this number, so the added utility of more experts is uncertain. Finally, an important limitation of this study is the use of only four ACL injuries, which do not represent the full spectrum of ACL injury, and do not take into account diversity of BMI somatotype, or other physical features. Despite these limitations, this study represents the first effort to create a standardized diagnostic approach to pediatric ACL tears and should serve as a framework for evaluation in this growing patient population.

## 5. Conclusions

As the incidence of pediatric ACL tears continues to climb, it is imperative to establish a standardized approach to the diagnostic evaluation of these injuries. Appropriately evaluating ACL injuries in skeletally immature athletes is critical for guiding the optimal management approach for the ACL injury while respecting the growing skeleton. In the absence of a standardized method to evaluate these patients, critical factors may be overlooked, and the same patient could be evaluated and treated differently between providers. In this Delphi study, we proposed a standardization of the diagnostic treatment approach derived from expert opinion that is applicable to all skeletally immature patients with ACL tears. This approach can serve as the framework for the evaluation of skeletally immature ACL patients to improve clinical decision making. Future work should focus on the expansion of an expert panel, including those individuals from other countries, as well as including a higher level of evidence in order to formulate a more concrete standard diagnostic evaluation pathway.

## Figures and Tables

**Table 1:** Consensus reached regarding pertinent factors in patient history with relevant sources of disagreement per vignette.

Vignette	Modal Response	Agreement	Consensus	Disagreement Sources
9F	Injury History	100	67%	
	Family History	92		
	Sports Participation	92		
	Current Symptoms	100		
10M	Injury History	100	83%	
	Family History	92		
	Sports Participation	92		
	Current Symptoms	100		
12F	Injury History	100	83%	Family History
	Sports Participation	92		
	Current Symptoms	100		
	Menarche	100		
13M	Family History	92	83%	
	Sports Participation	92		
	Current Symptoms	100		
	Injury History	100		

**Table 2:** Consensus reached in three vignettes regarding pertinent physical exam maneuvers with relevant sources of disagreement per vignette.

Vignette	Modal Response	Agreement	Consensus	Disagreement Sources
9F	Lachmann	100	67%	
	Pivot-shift	100		
	Collateral exam	100		
	Meniscal Exam	100		
	ROM	100		
	Ligamentous Laxity	100		
	Effusion	100		
10M	Lachmann	100	53%	
	Pivot-shift	100		
	Collateral exam	100		
	Meniscal Exam	100		
	ROM	100		
	Ligamentous Laxity	100		
	Effusion	100		
12F	Lachmann	100	75%	
	Pivot-shift	100		
	Collateral exam	100		
	Meniscal Exam	100		
	ROM	100		
	Ligamentous Laxity	100		
	Effusion	100		
13M	Lachmann	100	67%	Effusion
	Pivot-shift	100		
	Collateral exam	100		
	Meniscal Exam	100		
	ROM	100		
	Ligamentous Laxity	100		

**Table 3:** (**a**) Consensus reached in all vignettes regarding pertinent radiographic images. (**b**) Consensus reached regarding pertinent radiographic interpretation with relevant sources of disagreement per vignette.

Vignette	Modal Response	Agreement	Consensus	Disagreement Sources
(**a**)				
9F	Flexion	83	67%	
	Standing AP	100		
	Lateral	100		
	Long-leg Alignment	92		
	Bone Age Wrist Films	83		
10M	Flexion	83	67%	
	Standing AP	100		
	Lateral	100		
	Long-leg Alignment	92		
	Bone Age Wrist Films	83		
12F	Flexion	93	67%	
	Standing AP	100		
	Lateral	100		
	Long-leg Alignment	92		
	Bone Age Wrist Films	92		
13M	Flexion	83	67%	
	Standing AP	100		
	Lateral	100		
	Long-leg Alignment	92		
	Bone Age Wrist Films	92		
(**b**)				
9F	Tibial Slope	100	67%	
10M	Tibial Slope	100	67%	Mechanical Axis Alignment
	Physeal Status	100		
12F	Tibial Slope	100	75%	
13M	Tibial Slope	100	67%	Mechanical Axis Alignment
	Physeal Status	100		

**Table 4:** Summative Delphi results with agreement (* ≥66%) responses and topic of controversy (#). The final column presents our proposed standardized diagnostic pathway for patients with pediatric ACL injuries.

Diagnostic Evaluation Technique	9F	10M	12F	13M	All
Patient History					
Injury History	*	*	*	*	*
Family History	*	*	#	*	*
Sports Participation	*	*	*	*	*
Current Symptoms	*	*	*	*	*
Menarche			*		*
Physical Exam					
Lachmann	*	*	*	*	*
Pivot-shift	*	*	*	*	*
Collateral Exam	*	*	*	*	*
Meniscal Exam	*	*	*	*	*
ROM	*	*	*	*	*
Ligamentous Laxity	*	*	*	*	*
Effusion	*	*	*		*
Radiographic Sequence					
Flexion	*	*	*	*	*
Standing AP	*	*	*	*	*
Lateral	*	*	*	*	*
Long-leg Alignment	*	*	*	*	*
Bone Age Wrist Films	*	*	*	*	*
Radiographic Interpretation					
Tibial Slope	*	*	*	*	*
Physeal Status		*		*	*
Mechanical alignment/leg lengths	#	#	#	#	*
Skeletal age	#	#	#	#	*
Cross-Sectional Imaging					
MRI	*	*	*	*	*
Cross-Sectional Measurement					
None	*	*	*	*	*
Ancillary Studies					
None	*	*	*	*	*

## Data Availability

The data presented in this study are available on request from the corresponding author. The data are not publicly available due to institutional protocol.
